# Supplementary material for: Application of a ImageJ-Based Method to Measure Blood Flow in Adult Zebrafish and Its Applications for Toxicological and Pharmacological Assessments
Source: Biology (Basel). 2025 Jan 10;14(1):51. doi: 10.3390/biology14010051 (PMC11763070; doi:10.3390/biology14010051)
Supplement: Supplementary file 1 [file biology-14-00051-s001.zip › Figure S1, 2, 3.pdf]

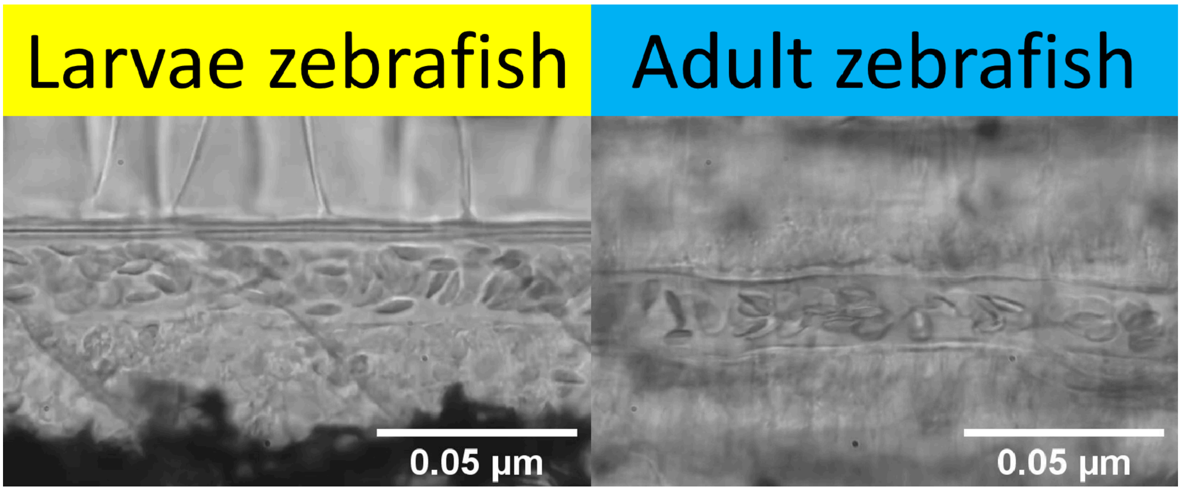

Figure S1. Comparison of blood shaped of zebrafish during larvae (3 days post fertilization) and adult stages (>5 months old)

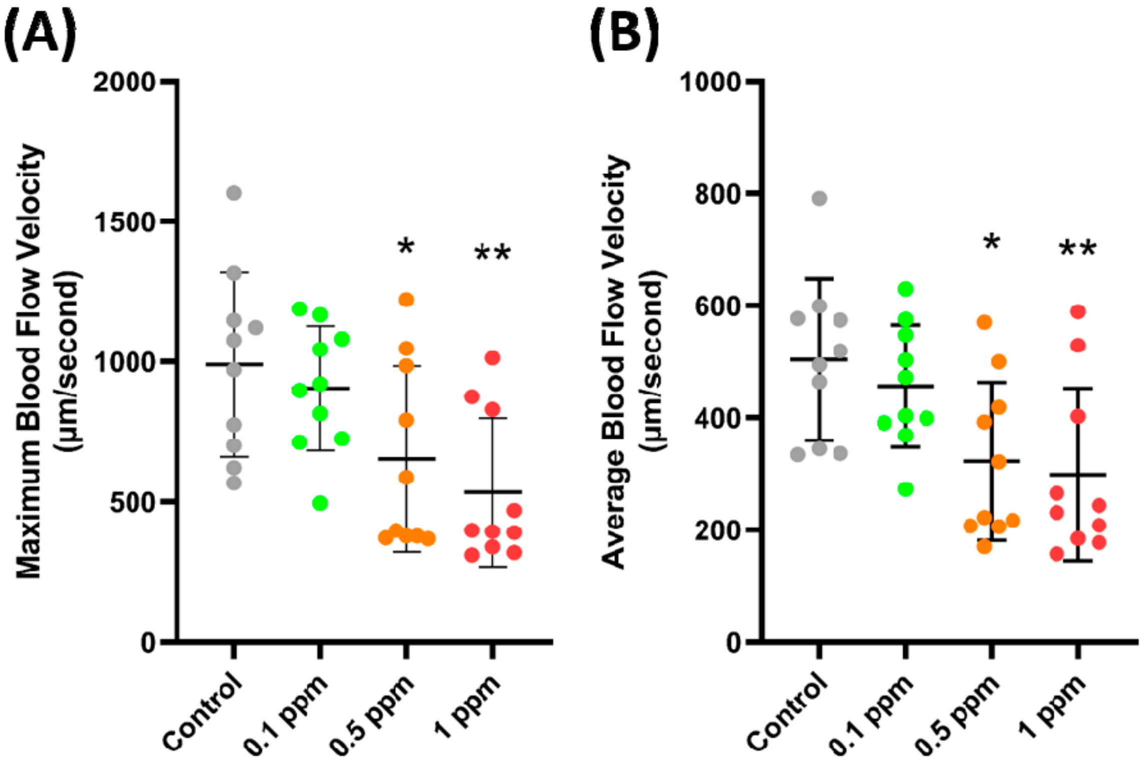

Figure S2. Maximum (A) and average (B) blood flow velocity of zebrafish after 24 hours exposed to carbofuran. The statistical difference was calculated using Ordinary One-Way ANOVA with Dunnet's multiple comparison test (*p<0.05, ***p<0.001) (*n* Control = 10, *n* 0.1 ppm = 10, *n* 0.5 ppm = 10, *n* 1 ppm = 10).

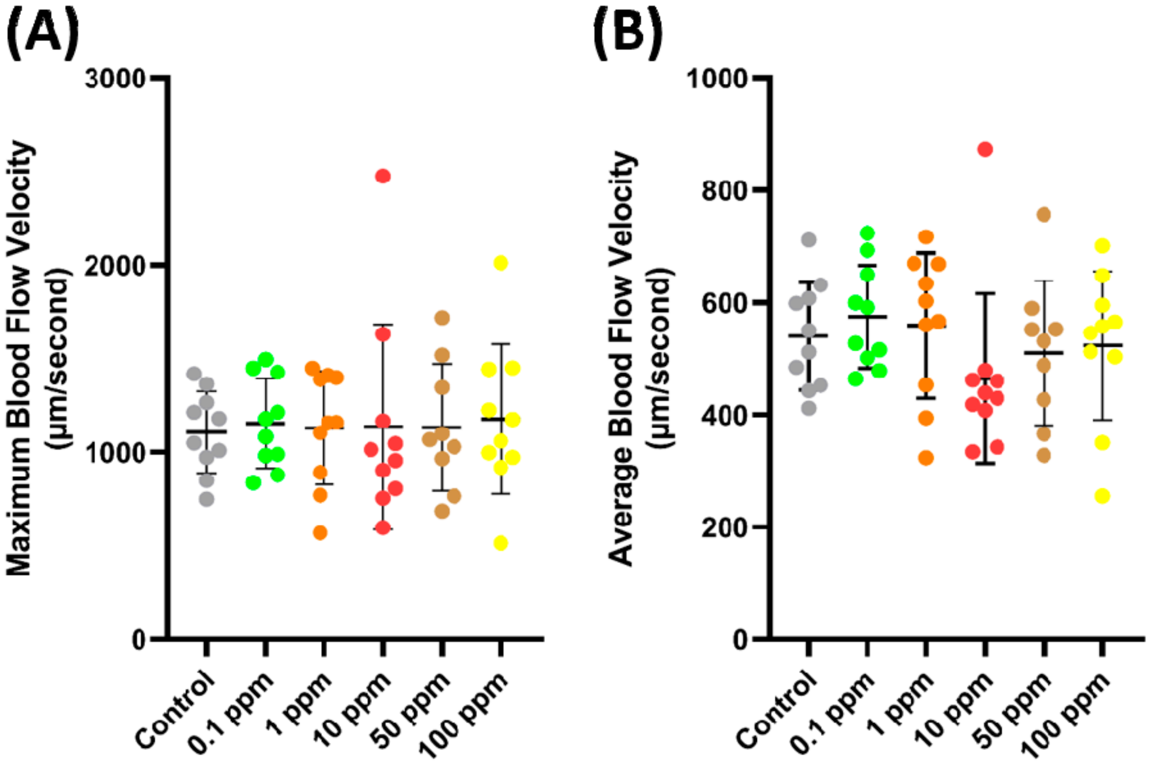

Figure S3. Maximum (A) and average (B) blood flow velocity of zebrafish after 24 hours exposed to copper oxide. The statistical difference was calculated using Ordinary One-Way ANOVA with Dunnet's multiple comparison test (*p<0.05, ***p<0.001) (*n* Control = 10, *n* 0.1 ppm = 10, *n* 1 ppm = 10, *n* 10 ppm = 10, *n* 50 ppm = 9, *n* 100 ppm = 10).
