# Supplementary material for: Application of a ImageJ-Based Method to Measure Blood Flow in Adult Zebrafish and Its Applications for Toxicological and Pharmacological Assessments
Source: Biology (Basel). 2025 Jan 10;14(1):51. doi: 10.3390/biology14010051 (PMC11763070; doi:10.3390/biology14010051)
Supplement: Supplementary file 1 [file biology-14-00051-s001.zip › Supplementary Table.pdf]

Table S1. p-value of maximum and average blood flow velocity at different temperature

Temperature	p-value	
	Maximum Velocity	Average Velocity
32°C vs. 26°C	0.9325	0.5814
32°C vs. 20°C	0.9991	0.0211
32°C vs. 15°C	<0.0001	<0.0001
26°C vs. 20°C	0.8857	0.347
26°C vs. 15°C	<0.0001	<0.0001
20°C vs. 15°C	<0.0001	0.0053
